# Prophylactic effect of angiotensin receptor blockers in children with genetic aortopathies: the early bird catches the worm

**DOI:** 10.1007/s00392-023-02221-4

**Published:** 2023-05-09

**Authors:** J. Olfe, J. J. Kanitz, V. C. Stark, F. Stute, Y. von Kodolitsch, D. Biermann, M. Huebler, R. Kozlik-Feldmann, T. S. Mir

**Affiliations:** 1 Children´s Heart Clinic, University Medical Center Hamburg-Eppendorf, Martinistrasse 52, 20246 Hamburg, Germany; 2 German Aortic Center, University Medical Center Hamburg-Eppendorf, Hamburg, Germany; 3 German Centre for Cardiovascular Research (DZHK), Partner Site/Kiel/Lübeck, Hamburg, Germany

**Keywords:** Marfan, Children, Sartan, Betablocker, Genetic aortopathies

## Abstract

**Aims:**

In genetic aortopathies (GA) particular attention is paid to aortic root dilatation which has an impact on morbidity and mortality. This study focuses on the effects of therapy with angiotensin-II-receptor-blockers (ARB) or beta-blockers (BB) on aortic root growth and the question which therapy should be initiated at which dosage and at what age.

**Methods:**

Since 1998 we diagnosed 208 patients with GA (170 FBN-1). 81 patients between 5 months and 18 years receiving either ARB or BB therapy were included. We retrospectively analyzed the progression of the dilatation of Sinus Valsalva aortae (SV) using calculated z-scores before and after therapy initiation and compared BB and ARB treatment.

**Results:**

Both ARB and BB (p < 0.05) therapy showed significant improvement in aortic root growth, while the effect is significantly more pronounced in ARB (p < 0.01) independent of age and genetic cause. A detailed comparison of the two drug groups showed a more sustained effect in limiting the progression of the dilatation of the aortic root in patients treated with ARB. Progression of dilatation of the SV was significantly lower in children treated with ARBs compared to BB (delta z-score, p < 0.05). In addition, ARBs were better tolerated and had a significantly lower discontinuation rate (3%) compared to BB (50%) (p < 0.01). Independently of age at initiation all children and adolescents were able to reach the target dose under ARB.

**Conclusion:**

We demonstrated a significant change in both treatment options, with the effect of ARB being more pronounced while being better tolerated throughout the treatment period.

## Introduction

Marfan syndrome (MFS) is an autosomal dominant inherited disease of connective tissue caused by a variation in the fibrillin-1-gene (FBN-1) in 90% of the cases [1, 2]. It phenotypically manifests predominantly in the skeletal, ocular, and cardiovascular systems, as well as the skin, lung, and dura [3–5].

Cardiovascular manifestations, such as dilatation of the Sinus Valsalva (SV) up to aortic dissection, largely determine morbidity and mortality [6, 7]. Aortic dissections are responsible for the majority of premature mortality in MFS patients [6] and the risk of aortic dissection increases steeply from adolescence. Early prophylactic treatment of dilatation of the aortic root is therefore of central importance in terms of prevention of morbidity and mortality [8].

Halpern et al. suggested as early as 1971 that blockade of β-adrenergic receptors might reduce the risk of aortic dissection [9]. Until now the therapeutic goal is to prophylactically reduce the hemodynamic shear stress of the aortic wall and thus delay or even prevent aneurysm formation [10, 11]. It has been shown in both adult and pediatric studies that beta-blockers (BB) lead to a significant reduction in aortic diameter and reduce the risk of occurrence of cardiovascular complications [10, 12]. However, there are other study results that could not reflect this effect with heterogeneous results. Data from long-term studies with BB therapy in pediatric Marfan patients are not yet known. Especially in pediatric patients tolerability of beta blockers is poor and rates of discontinuation of therapy are high. Therefore in real-world patient management, the theoretical effect of BB can often not be established.

Recent preclinical models in a mouse model of Marfan syndrome have shown that variation in the FBN-1 gene is associated with excessive activation of transforming growth factor-beta (TGFβ), which in turn contributes to the phenotypic expression of MFS, including SV dilation. Mouse models treated with angiotensin II receptor blockers (ARB) showed significantly lower rates of SV growth compared with their untreated littermates, suggesting a potential therapeutic benefit of ARB [13] for the first time. ARB therapy also reduced SV growth rate in studies of MFS patients [14, 15] and has been associated with better clinical outcomes in adults [16]. There are studies in the pediatric setting that have demonstrated a positive effect of ARB on SV z-score as well [14, 17]. In addition, ARB therapy has been shown to result in fewer side effects and better tolerability [18]. However, there are contradictory study results with this group of drugs. There are also pediatric studies that could not present the expected effect of ARB therapy [19, 20]. The mice treated in the animal models received an extremely high dosage of ARB that cannot be reached in humans and thus the therapeutic effects shown in human studies were always disappointing. Nevertheless, it shows that a higher dosage of ARB might lead to a more pronounced effect. In children, the tolerability of ARB is good and high doses up to 3 mg/kg bodyweight are easily accepted even in small toddlers. Therefore in real life target doses of ARBs are reached much easier than in BB treatment.

**Fig. 1:**
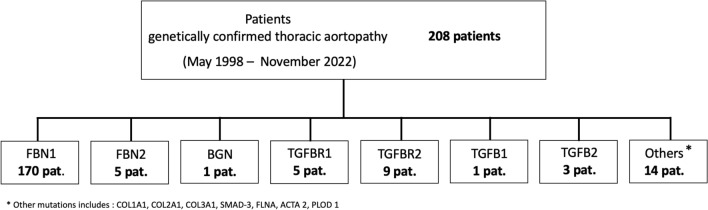
Number of genetic variants in patients with genetically confirmed thoracic aortopathy

Since its presentation in 1971, BB therapy has been considered the first-line therapy for MFS. Due to new findings, ARB have gained importance regarding the therapy of MFS. This leads to the question of which drug is the better choice for therapy in children. In the mouse model, promising results indicated that ARB have a stronger effect than BB [13], but several clinical trials could not confirm that ARB are better than BB [21]. However, it has already been shown that the younger the patient is at the initiation of therapy, the more effective the effect of the drug is [10]. For this reason, it seems of great importance to start the optimal therapy at an early age to achieve the best possible benefit. Moreover, tolerability is key in pediatric medical treatment and only drugs that are given regularly and in high doses by the parents can be effective. Therefore, at this point, the question arises as to which therapy should be initiated at which dosage and at what age (Fig. 1)

## Patients, material and methods

Between May 1998 and November 2022, 208 patients with a genetically confirmed aortopathy were examined in the special consultation of Children´s Heart Clinic of the University Medical Center Hamburg-Eppendorf. 170 patients were carriers of an FBN-1-mutation and therefore have a genetically confirmed Marfan syndrome.

**Fig. 2:**
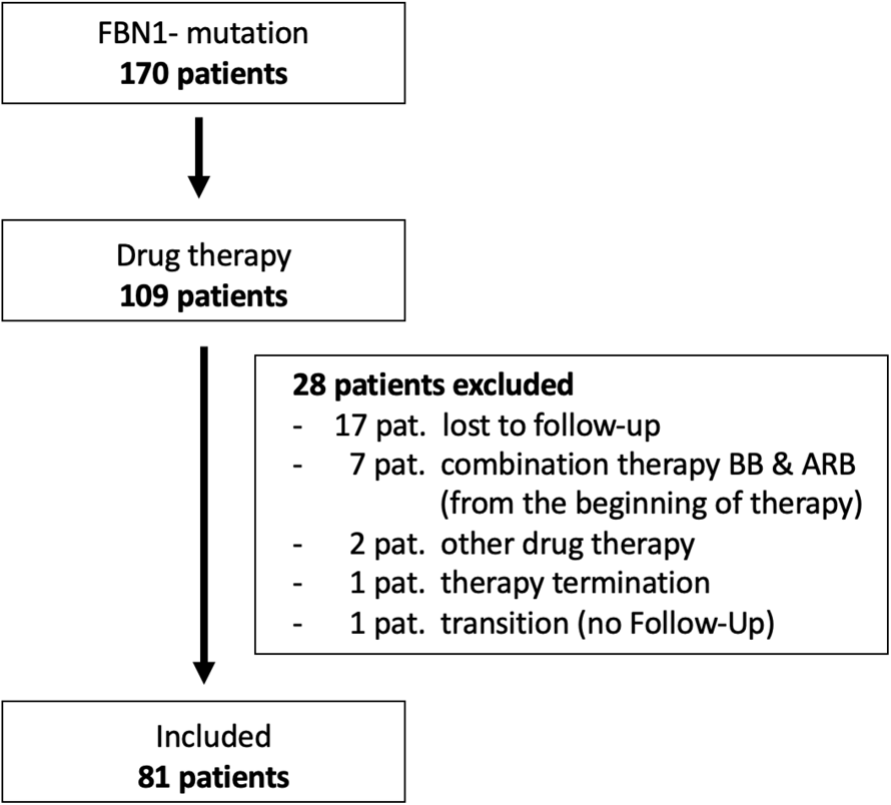
Patient population and exclusion criteria

Retrospectively, 81 patients were included in the analysis who were treated with BB, ARB, or who were switched to a combination of both drugs during therapy. All patients included in this study (n = 81) had a genetically confirmed MFS with a variant in the FBN-1 gene and have not had previous aortic surgery (Fig. 2). Further inclusion criteria were at least 2 visits with at least 3 months intervals.

Until 2010, when first reports about the effect of ARB an aortic growth were published, all patients were treated with BB as first-line therapy and no escalation of therapy to ARB was known and thus not performed. From 2010 onwards the first patients were started on ARB as first-line therapy. In those with insufficient response to ARB treatment, escalation to double-therapy with ARB + BB was performed. From 2010s onwards, patients having side effects due to treatment with BB were switched to ARB alone.

For this restrospective analysis patients were divided into two groups according to their therapy. 75 patients received ARB therapy which was started at a mean age of 8.1 ± 4.7 years. 14 patients received therapy with BB. They started therapy at a mean age of 9.9 ± 5.6 years.

A separate group is represented by 16 patients who changed between drugs during the follow-up period. This group includes only those patients in whom the previous therapy, as well as the newly selected therapy, consisted of BB, ARB, or combination therapy of these two drug groups. Other drug groups were excluded. These patients were assigned to the current therapeutic group according to their treatment. They are therefore present in multiple groups (Tables 1, 2, 3, 4).

**Table 1:** Demographic data before initiation of therapy

	ARB	BB	p-value
Number (n)	75	14	
Gender (female)	33/ 44%	6/ 43%	
Age at treatment initiation (age in years)	8.1 ± 4.8	10 ± 5.6	0.179
Follow-up period (in months)	7.5 ± 5.4	8.8 ± 5.8	0.409

*ARB* angiotensin receptor blocker, *BB* beta blocker

**Table 2:** Therapy change and timing

	Number (n)	Age (years)
BB—> ARB	6/16	13.5 ± 5.8
BB—> BB + ARB—> ARB	4/16	9.2 ± 1.8 10.2 ± 1.9
BB—> BB + ARB	1/16	16.6
ARB—> BB + ARB	5/16	8.5 ± 5.3

*ARB* angiotensin receptor blocker, *BB* beta blocker

**Table 3:** *z-scores* of the aortic annulus, Sinus Valsalva Aortae, and sinotubular junction before and after initiation of angiotensin receptor blocker (ARB) and beta blocker (BB) therapy

	ARB	BB	p-value
Before treatment			
Z-Score annulus	1.41 ± 1.19	1.51 ± 1.36	0.814
Z-Score Sinus Valsalva	1.88 ± 1.03	2.66 ± 1.59	0.101
Z-Score sinotubular junction	0.96 ± 0.97	1.74 ± 1.06	< 0.05 (0.022)
At follow-up			
Z-Score annulus	1.20 ± 1.11	2.10 ± 1.28	< 0.05 (0.016)
Z-Score Sinus Valsalva	1.68 ± 1.01	2.86 ± 1.50	< 0.05 (0.013)
Z-Score sinotubular junction	0.76 ± 0.92	2.16 ± 1.31	< 0.01 (0.000)
Difference*			
Z-Score annulus	-0.21 ± 0.95	0.59 ± 0.88	< 0.05 (0.010)
Z-Score Sinus Valsalva	-0.20 ± 0.41	0.20 ± 0.33	< 0.01 (0.001)
Z-Score sinotubular junction	-0.20 ± 0.75	0.42 ± 1.01	< 0.05 (0.021)

ARB angiotensin-receptor-blocker (n = 75), *BB* betablocker (n = 14)

Age in years (average): ARB = 8,1 / BB = 10 / p = 0.179

*between z-score values before therapy initiation and follow-up after therapy initiation

To avoid selection bias in the latter group of patients, we performed a subgroup analysis considering only the first drug given at therapy initiation (Table 4).

**Table 4:** Sub-group analysis (Inclusion of only ARB as first-line therapy)

	ARB	BB	p-value
Before treatment			
Z-Score annulus	1.32 ± 0.98	1.51 ± 1.36	0.588
Z-Score Sinus Valsalva	1.85 ± 0.94	2.66 ± 1.59	0.086
Z-Score sinotubular junction	0.88 ± 0.89	1.74 ± 1.06	< 0.01 (0.008)
At follow-up			
Z-Score annulus	1.18 ± 0.94	2.10 ± 1.28	< 0.01 (0.006)
Z-Score Sinus Valsalva	1.62 ± 0.90	2.86 ± 1.50	< 0.01 (0.009)
Z-Score sinotubular junction	0.72 ± 0.86	2.16 ± 1.31	< 0.01 (< 0.001)
Difference*			
Z-Score annulus	− 0.14 ± 0.89	0.59 ± 0.88	< 0.05 (0.014)
Z-Score Sinus Valsalva	− 0.23 ± 0.40	0.20 ± 0.33	< 0.01 (< 0.001)
Z-Score sinotubular junction	− 0.17 ± 0.74	0.42 ± 1.01	< 0.05 (0.030)

*between z-score values before therapy initiation and follow-up after therapy initiation

*z-scores* of the aortic annulus, Sinus Valsalva Aortae, and sinotubular junction before and after initiation of angiotensin receptor blocker (ARB) and beta blocker (BB) therapy

*ARB* Angiotensin-receptor-blocker (n = 66), *BB*  Betablocker (n = 14)

Age in years (average): ARB = 7.57 / BB = 9.98 / p = 0.085

In this analysis, the effect of BB and ARB on aortic growth was investigated. For this purpose, the z-scores before the start of therapy were compared with the values of the first visit after initiation of therapy (at least 3 months in between). Follow-up duration averaged 7.5 ± 5.4 months in the ARB group and 8.8 ± 5.8 months in the BB group. In patients who changed their therapy group, the difference between the last follow-up with old medication and the first follow-up with the new (at least 3 months apart) was considered.

Detailed evaluation of every patient include a physical examination based on the Ghent Nosology using the Revised Ghent Criterias (RGC) [22, 23] and a pediatric cardiac examination including echocardiography and electrocardiogram (ECG). If indicated and with parental consent, genetic analysis was performed. In addition, magnetic resonance imaging (MRI) was performed at regular intervals according to clinical criteria if indicated. Echocardiographic follow-up was performed at intervals between 3 and 12 months.

The echocardiographic examination is performed in the supine or left lateral decubitus position. The aortic root is viewed in the parasternal long axis and its diameter is measured at three levels (annulus, sinotubular junction, and SV) on 2D- images using the leading-edge-to-leading-edge technique in end-diastole. For all parameters, a calculation of the associated z-score is performed. For the annulus, sinotubular junction, and SV the z-scores were calculated according to Pettersen et al. [24].

Drug treatment was initially started in case of SV dilatation outside the normal range of a z-score of ± 2 described by Pettersen et al. [24, 25]. In November 2002, BB therapy was prescribed for the first time. On average, we started BB therapy at a SV z-score of 2.66 ± 1.59. For BB therapy, the standard doses corresponding to the drug were chosen. The initial dose for therapy with BB was 1 mg/kg bw/day every 6–12 h and was increased up to 1–2 mg/kg bw/day during follow-up.

Due to new recommendations, early prophylaxis was increasingly started already in cases of mild to moderate SV dilatation during the analyzed period [10, 26]. In this case, factors such as a rapidly progressing dilatation of the SV play a role in the choice of therapy initiation. The first therapy with ARB was in October 2008. The average SV z-score in the ARB therapy group was 1,88 ± 1,03 when therapy was started. The initial dose with ARB (Valsartan) is 1 mg/kg bw/d in two single doses and was increased to a maximum of 3 mg/kg bw/d depending on tolerability. The dose is adjusted within the follow-up controls according to the progression of dilatation and body weight. If drug intolerance occurred, therapy was changed or terminated according to clinical guidelines.

## Statistical analysis

The two-tailed t-test for independent variables was used to evaluate and test the results for significance.

For comparison of aortic root z-scores before initiation of treatment and follow-up under treatment within a group, the paired-samples t-test was used. Categorical data were compared with the chi-square test and Fisher's exact test when needed. All values are expressed as mean ± SD. P values < 0.05 were considered statistically significant.

The collected data were archived using the database program Filemaker Pro version 11.0v3. Statistical analysis, as well as presentation of results, was performed using Excel 16.50 and IBM SPSS Statistics version 17.

The study was designed, performed and controlled according to current guidelines of Good Clinical Practice and approved by the local ethics committee.

## Results

Of 170 patients who had FBN-1 associated Marfan syndrome, we were able to include 81 patients in the analysis. In 16 patients, the drugs were switched during the course of therapy. They are represented in several groups. Two groups were formed according to their medication. 75 patients were taking ARB and 14 patients were receiving BB therapy.

In the group of patients in whom the therapeutic scheme was switched, 6/16 patients were switched from BB to ARB at 13.5 ± 5.8 years due to side effects or intolerance. In 5/16 patients, therapy was discontinued from ARB to a combination of BB & ARB due to rapidly dilating SV at age 8.5 ± 5.3 and one patient changed from BB to a combination of BB & ARB at age 16.6. Another 4/ 16 patients underwent conversion from BB to a combination of BB & ARB (at age 9.2 ± 1.8) to final therapy with ARB (at age 10.2 ± 1.9).

Before therapy initiation, the ARB group had a mean z-score in the aortic annulus of 1.41 ± 1.19 in the Sinus Valsalva 1.88 ± 1.03 and in the sinotubular junction 0.96 ± 0.97. In the group of children treated with BB a mean z-score in the aortic annulus of 1.51 ± 1.36, in the Sinus Valsalva of 2.66 ± 1.59, and in the sinotubular junction of 1.74 ± 1.06 before therapy initiation was observed.

During BB treatment, z-scores increased to 2.10 ± 1.28 (p-value < 0.05 (0.049)) in the aortic annulus, 2.86 ± 1.50 (p-value < 0.05 (0.043)) in the Sinus Valsalva and 2.16 ± 1.31 (p-value 0.216) in the sinotubular junction.

After initiation of therapy with ARB, the mean z-score values decreased to 1.20 ± 1.11 (p-value 0.061) in the aortic annulus, 1.68 ± 1.01 (p-value < 0.01 (0.000)) in the SV and 0.76 ± 0.92 (p-value < 0.05 (0.029)) in the sinotubular junction.

Between the two time points, patients treated with BB showed an increase of z-scores values of 0.20 ± 0.33 (p-value < 0.05) in the SV while patients treated with ARB showed a decrease of -0.20 ± 0.41 (p-value < 0.01) in the z-score of SV. Comparison of the z-score differences between the two drug groups showed a p-value < 0.01.

## Discussion

The present study compares the influence of BB and ARB on the growth of the aorta of MFS patients in childhood. Dilatation of the aortic root persists in the majority of patients with MFS and many patients develop dilatation of the SV in early childhood. It is considered the best predictor of adverse cardiovascular outcomes [27]. Accordingly, early initiation of medical therapy plays an important role in influencing morbidity and mortality. Because of the current still inconclusive data in the pediatric setting, the question arises, on the one hand, which drug is best suited for early therapy and on the other hand at what age and at which dose therapy should be started.

We retrospectively analyzed the aortic parameters before and after therapy initiation between the two drug groups BB and ARB in patients with genetically confirmed MFS. The initial dose for therapy with BB (predominantly Metropolol) was 1 mg/kg bw/day every 6–12 h and was increased up to 1–2 mg/kg bw/day during follow-up, according to dosing guidelines. For therapy using ARB (predominantly Valsartan), the initial dosage was 1 mg/kg bw/d in two single doses and was increased up to a high dose range of max. 3 mg/kg bw/d depending on tolerability. ARB therapy showed a significantly greater decrease in SV z-scores than BB. Looking at the z-scores of the SV parameter, we see that the z-score decreases by 0.20 on average under ARB therapy, while it increases by 0.20 under BB therapy.

Before therapy initiation the diameter of the SV was statistically not different between both groups. but first-line therapy in our department was changed in 2010 so ARBs were predominantly used instead of BB since then. Under the notion that ARBs have a causal and thus prophylactic effect on connective tissue integrity, especially in childhood therapy with ARB was started already for moderate dilatations and especially at an earlier age.

Table 1 shows that there was no statistical age difference between the two groups. Nevertheless,we started ARB therapy on average earlier at an age of 8 years and BB therapy at an age of 10 years. Our youngest patient received ARB therapy initiation at an age of 5 months compared to an initiation age of 2.5 years with BB.

QueryOur current study results consolidate previously published data and show that therapy with both BB and ARB leads to a significant change of the z-score at the SV. They also provide indications that therapy with ARBs when started prophylactically leads to a stronger effect at the SV compared with beta-blockers. Early initiation of therapy in the form of prophylaxis also appears helpful because of the mechanism of action of ARBs, which presumably interferes causative in the pathology. ARBs act via selective blockade of the angiotensin-II-type-1-receptor within the renin–angiotensin–aldosterone system [28, 29] and additionally cause a clinically relevant reduction in TGF-β signaling [13, 29].

Another argument for early therapy comes from the study by Lacro et al., in which it was shown that when patients are younger at the initiation of therapy with ARBs, a greater decrease in aortic root z- score is observed over time [18]. The results of our current study also provide indications for this statement. Compared with other pediatric studies, we started prophylactic therapy with ARB much earlier in terms of age (around 3 years earlier) [15, 18] as well as in terms of z-score. Overall a strong effect of ARB in terms of the dilation of SV could be shown.

Since two studies on the benefit of BB were published by Taherina et al. in 1993 and Shores et al. in 1994, they have been used as the standard of care for MFS [12, 30]. However, there is no unanimous opinion on the treatment of progressive aortic dilatation because of the inconsistent study results. The benefit of BB is controversial because of studies with conflicting results. For example, the study by Selamet-Tierney et al. failed to demonstrate the beneficial effect of BB therapy [31]. Furthermore, BB are less well tolerated, particularly in children, and therapy is more frequently discontinued because of side effects. It was shown in the study by Lacro et al. that symptoms such as nausea and dizziness when standing significantly increased with BB [18]. In our study therapy with ARB was also better tolerated overall. Within the follow-up period, two patients in the ARB therapy group (n = 75) discontinued therapy due to depression symptoms in one and increased occurrence of syncope in another. No patient was switched from ARB to BB because of side effects. In the BB therapy group (n = 14), there was one complete discontinuation of therapy and 6 patients were switched from BB to ARB in the course of their therapy due to intolerances/side effects. In order to control for selection bias due to discontinuation or switch of therapies during the study period, we did a subgroup analysis only looking at patients being treated with ARB alone (Table 4). The effect on the SV remained the same.

With the publication by Habashi et al. and the effect of ARB on mouse models [13] another class of drugs has been suggested to play an important role in the therapy of MFS.

A recent study of exclusively pediatric patients by Pees et al. demonstrated a significant reduction in aortic root z-score with ARB (losartan) treatment within the first 36 months. The reduction in z-score was associated with an improvement in aortic elasticity. There was evidence that aortic tissue stiffness had a predictive role and that low scores predicted a better response to therapy. However, it was also observed that the effect of ARB therapy attenuated after 36 months, as did the effect of aortic tissue stiffness [15]. In studies of adult patients long-term ARB treatment was associated with better clinical outcomes [16].

Several studies have already looked at the comparison of ARB and BB and here contradictory results emerged, too. A recently published meta-analysis showed that ARBs reduce the rate of aortic root dilatation by about one-half in patients with Marfan syndrome. When comparing ARB and BB the effects of the two groups of drugs turned out to be similar [32]. Also, a review by Singh et al. found no significant differences between BB and ARB [10]. One study named in the review, by the Pediatric Heart Network [18], was primarily concerned with the long-term effects of therapy over a 3-year period. Patients with z-scores < 3 were excluded from the study [18]. Losartan was chosen as the drug for ARB therapy. In contrast, we predominantly use valsartan. There are minor pharmacodynamic and -kinetic differences between the two ARBs. The extent to which these differences are clinically relevant remains to be elucidated [33]. Even these minor differences may be related to the greater effect on SV growth rate.

**Fig. 3:**
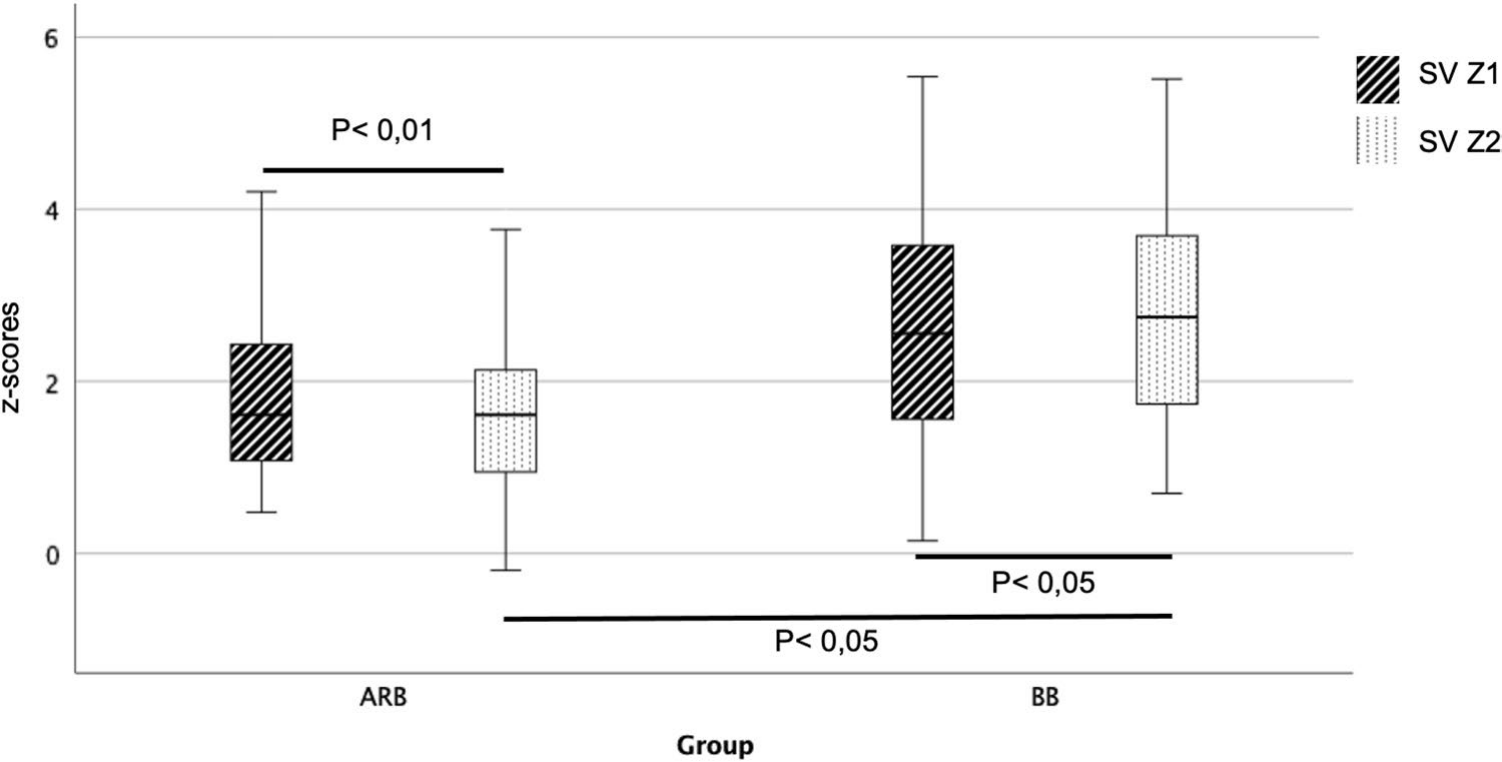
Change in Sinus Valsalva (SV) z-score before and after initiation of therapy with angiotensin receptor blocker (ARB) and beta blocker (BB) at time Z1 (before initiation of therapy) and Z2 (after initiation of therapy)

**Fig. 4:**
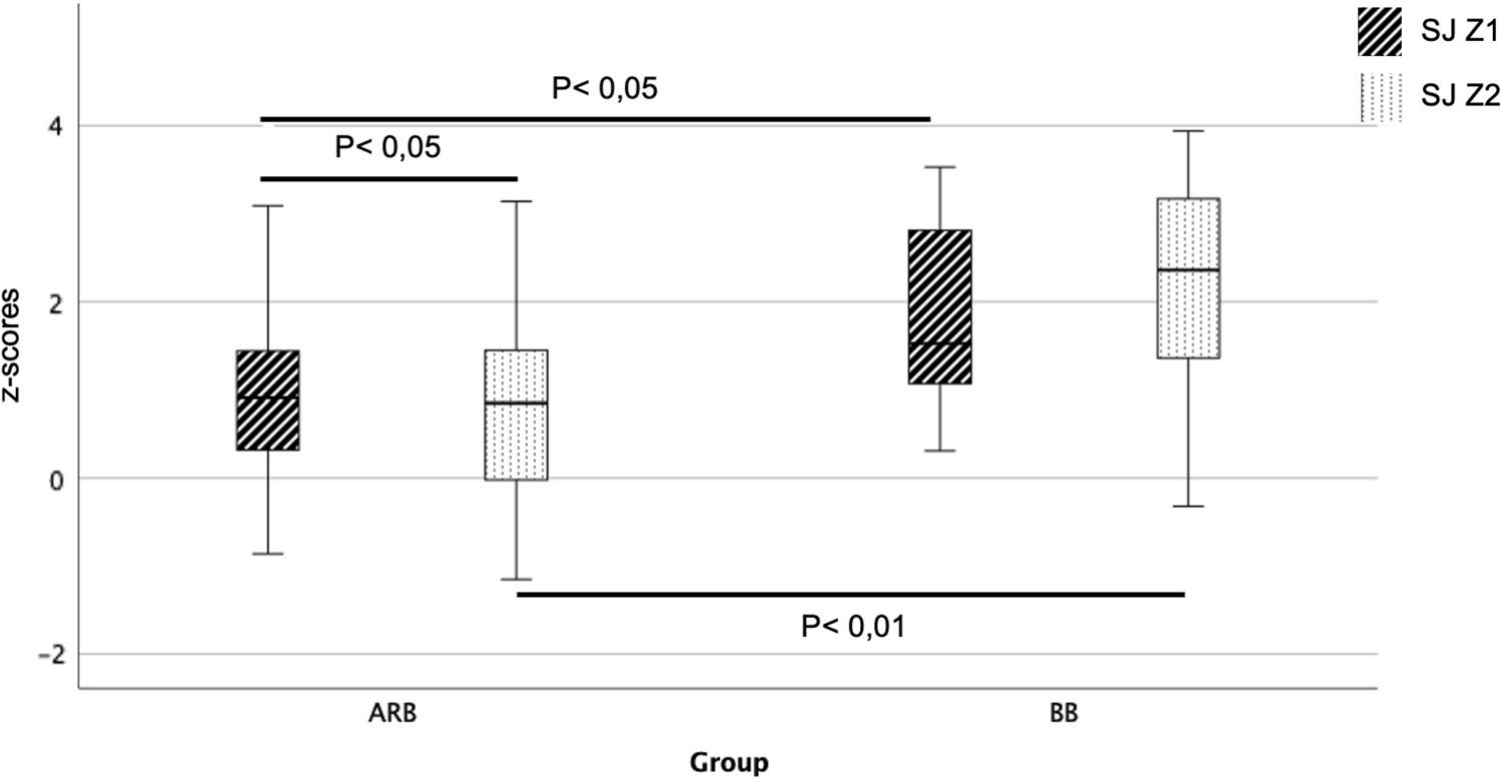
Change in sinotubular junction (SJ) z-score before and after initiation of therapy with angiotensin receptor blocker (ARB) and beta blocker (BB) at time Z1 (before initiation of therapy) and Z2 (after initiation of therapy)

**Fig. 5:**
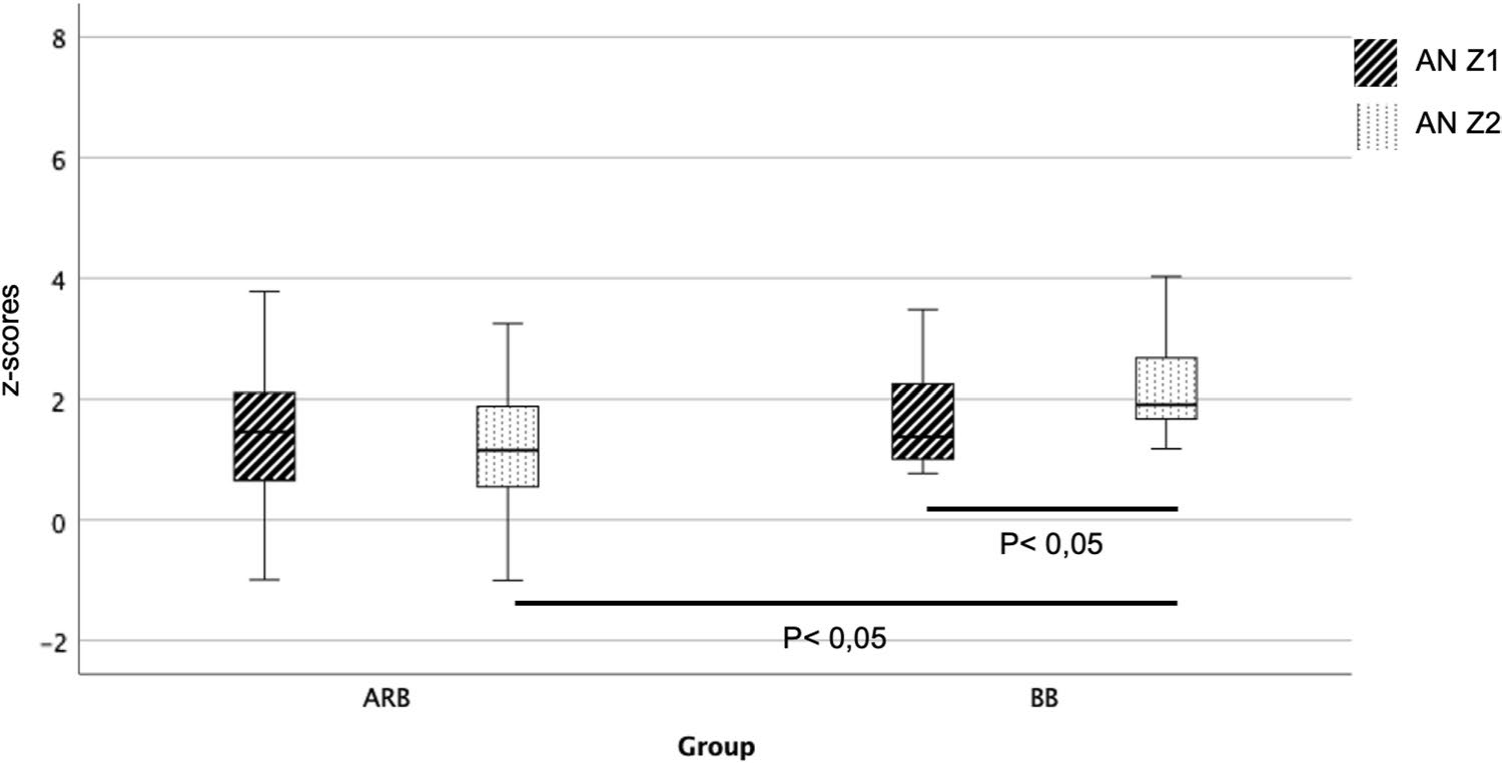
Change in aortic annulus (AN) z-score before and after initiation of therapy with angiotensin receptor blocker (ARB) and beta blocker (BB) therapy at time Z1 (before initiation of therapy) and Z2 (after initiation of therapy)

The Pediatric Heart Network demonstrated a significant reduction in SV with both ARB and BB therapy but overall did not show the expected advantage of ARB over BB. This could also be explained by the relatively low ARB doses. The initial dose in this study was 0.4 mg/kg bw/d and was increased to a maximum of 1.4 mg/kg bw/d [18]. In our study, a higher dosage of ARB of 1 mg/kg bw/d was already started at the beginning of therapy. The dose was increased up to a maximum of 3 mg/kg bw/d depending on tolerability and need. This high dosage may explain why our data show a stronger effect of ARB therapy in terms of preventing the progression of SV dilatation. However, data regarding the dose-dependent effect of ARB are not yet available (Figs. 3, 4, 5)

**Fig. 6:**
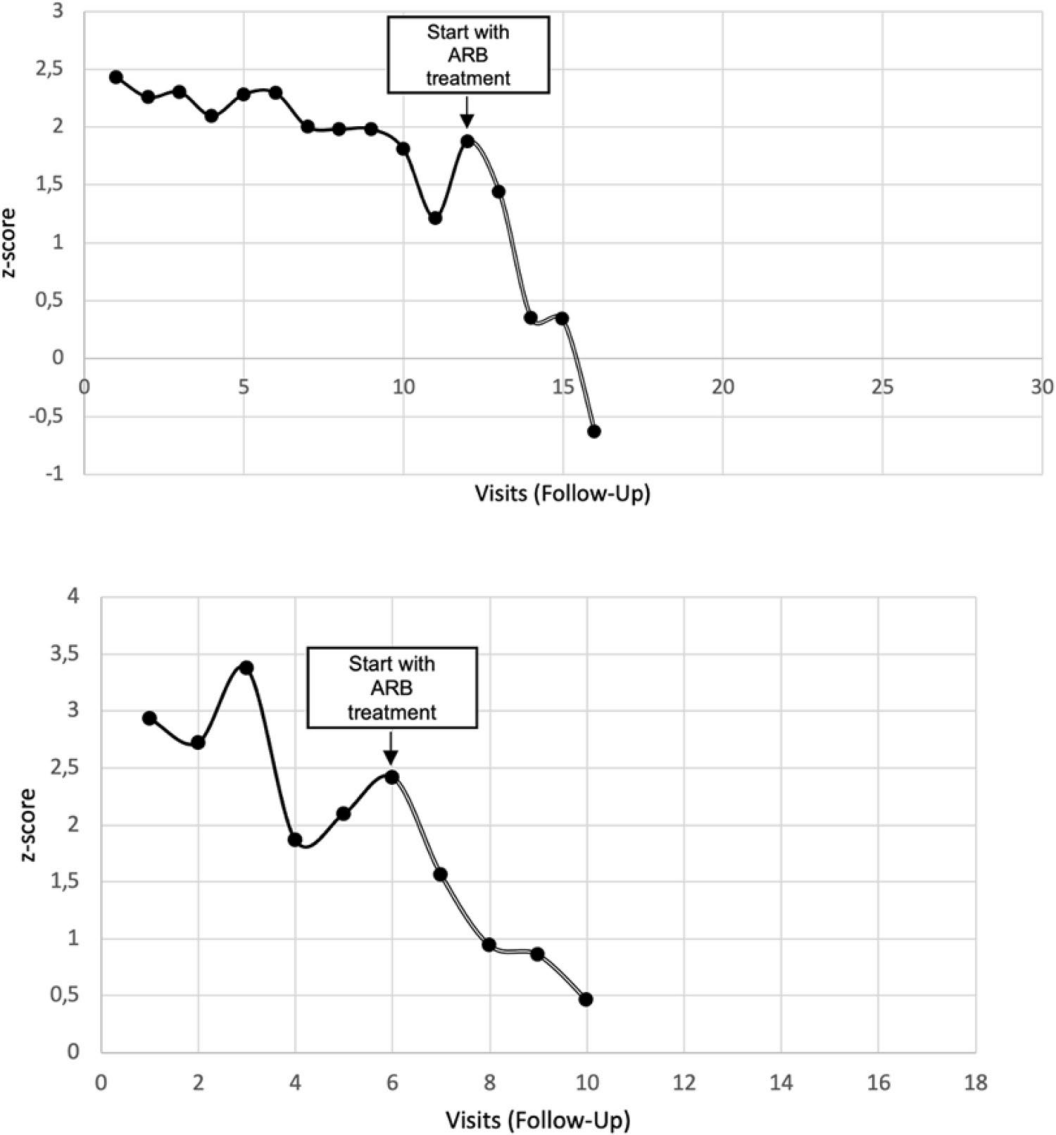
Illustration of the *z-scores* of two patients over several years, before and after initiation of ARB treatment, ARB = angiotensin receptor blocker

In this retrospective analysis, there are methodological differences from previously conducted studies that did not demonstrate differences between ARB and BB. This is an analysis with a purely pediatric patient population. To investigate the prophylactic effect, smaller z-scores starting at ± 0.15 were included as long as the inclusion criteria of an FBN-1 mutation and drug therapy were met. Only z-scores were considered; absolute numbers did not play a role in the analysis. As previously described, our patients were already treated with a higher initial ARB dose from baseline, which was increased as needed. Furthermore, a period of 7.5 (ARB) and 8.8 (BB) months on average was observed in this analysis, so the initial success of therapy after the start of treatment was particularly evident. Since BB are no longer first-line therapy in our institution for over 13 years, it has become difficult to analyse long-term data in both treatment groups. As an example of the long term effect of ARB in children Fig. 6 shows the effect of the initiation of ARB on the SV over the course of many visits.

There are also some limitations in the study we conducted. First, it is a retrospective evaluation of the data. Second, the study assumes patient compliance with regard to regular medication use which could not be controlled.

Our two medication groups differed in group strength (ARB n = 75 and BB n = 14). This can be explained by the fact that BB were used as first-line therapy before 2010. Therapy was indicated exclusively for severe aortic dilatation. With an improved understanding of the pathogenesis and mechanism of action of ARBs, early therapy has been started in moderate SV dilatation since 2010. Since then, we have predominantly used ARBs as first-line therapy. There are also differences in the age structure, as therapy is started at a younger age due to the switch to starting therapy earlier. Furthermore, a reduction in SV dilatation due to a lack of outcome data does not suggest that it also reduces the incidence of aortic root surgery, aortic dissection, death, or a combination of these events. Third, the influence of individual factors such as the severity of the pathology, age, and genotype on the effect of the drugs on the patient was not considered further and was not investigated in detail.

## Conclusion

Despite multiple studies, there is still disagreement about which drug to use, at what age, and at which dosage to start therapy in children with MFS. The present study provides evidence that therapy with ARB in children with Marfan syndrome started at an early age leads to a significant reduction in the diameter of the SV while being well tolerated. ARBs produced significantly stronger effects at initial therapy initiation compared with BB. Based on our study results compared with previously published data, there are indications that starting therapy at a young age as well as in high dosage may have an effect on the magnitude of the reduction in the z-score of the SV. For future studies, consideration of dose-dependent data would be of interest. Moreover, long-term side effects of either class of drugs initiated at a young age should be studied.

## Data Availability

The participants of this study did not give written consent for their data to be shared publicly, so due to the sensitive nature of the research supporting data is not available.
